# Searching High and Low: Prosodic Breaks Disambiguate Relative Clauses

**DOI:** 10.3389/fpsyg.2017.00096

**Published:** 2017-02-01

**Authors:** Lauren A. Fromont, Salvador Soto-Faraco, Emmanuel Biau

**Affiliations:** ^1^School of Speech Language Pathology and Audiology, University of Montreal, MontrealQC, Canada; ^2^Centre for Research on Brain, Language and MusicMontreal, Canada; ^3^Multisensory Research Group, Center for Brain and Cognition, Universitat Pompeu FabraBarcelona, Spain; ^4^Institució Catalana de Recerca i Estudis Avançats, BarcelonaSpain; ^5^Basic and Applied NeuroDynamics Lab, Maastricht UniversityMaastricht, Netherlands

**Keywords:** syntactic ambiguity, prosody, relative clause attachment, Spanish, syntactic parsing

## Abstract

During natural speech perception, listeners rely on a wide range of cues to support comprehension, from semantic context to prosodic information. There is a general consensus that prosody plays a role in syntactic parsing, but most studies focusing on ambiguous relative clauses (RC) show that prosodic cues, alone, are insufficient to reverse the preferred interpretation of sentence. These findings suggest that universally preferred structures (e.g., Late Closure principle) matter far more than prosodic cues in such cases. This study explores an alternative hypothesis: that the weak effect of prosody might be due to the influence of various syntactic, lexical-semantic, and acoustic confounding factors, and investigate the consequences of prosodic breaks while controlling these variables. We used Spanish RC sentences in three experimental conditions where the presence and position (following the first or second noun phrase) of prosodic breaks was manipulated. The results showed that the placement of a prosodic break determined sentence interpretation by changing the preferred attachment of the RC. Listeners’ natural preference for low attachment (in the absence of break) was reinforced when a prosodic break was placed after the first noun. In contrast, a prosodic break placed after the second noun reversed the preferred interpretation of the sentence, toward high attachment. We argue that, in addition to other factors, listeners indeed use prosodic breaks as robust cues to syntactic parsing during speech processing, as these cues may direct listeners toward one interpretation or another.

## Introduction

- *“One morning I shot an elephant in my pajamas… How he got into my pajamas, I don’t know”*

(*Groucho Marx, in Animal Crackers*, 1930) -

The sentence above illustrates how artists exploit prosodic modulations (here, the absence of explicit cues) to create a comic effect based on syntactic ambiguity. In daily social interactions, speakers use prosody to facilitate online spoken comprehension by cueing the correct parsing of sentences (Lehiste, 1973; Frazier et al., 2006). Prosody reflects subtle modulations that shape speech envelopes (e.g., silences, pitch accents) and allow the speaker to direct the listener’s attention toward relevant information. In addition, prosodic breaks are hypothesized to help listeners segment the signal into intonational phrases and facilitate decoding its syntactic structure. Some approaches argue for an immediate use of prosodic cues in sentence processing (anti-attachment hypothesis: Watson and Gibson, 2005), whereas others argue for a more global analysis of the prosodic structure (informative boundary hypothesis: Clifton et al., 2002). However, to what extent listeners rely on prosody to parse ambiguous sentences remains unclear. Such structures are well illustrated by the classic case of RC attachment ambiguity such as in (1) (for a review, see Fernández, 2003).

(1) Someone shot [the servant]_NP1_ of [the actress]_NP2_ [who was on the balcony]_RC_.
  (a) The servant was on the balcony.
  (b) The actress was on the balcony.

Relative clause ambiguous sentences yield two possible interpretations as the RC may attach with the first noun phrase (NP1), as in interpretation (a): “the servant” (HA) or, the second (NP2), as in (b): “the actress” (LA). On the one hand, the Late Closure Principle stated that across languages, listeners generally prefer the latter (LA) interpretation in case of syntactic ambiguity (Frazier, 1979; Fernández, 2003). On the other, Cuetos and Mitchell (1988) showed that Spanish speakers choose a HA interpretation instead, and that forcing an LA interpretation leads to increased reading times, suggesting higher processing costs (Carreiras, 1992; Carreiras and Clifton, 1999; see also Augurzky, 2005). While these findings led to very prolific cross-linguistic research, Gilboy et al. (1995) suggested that the observed variability is mostly due to construction types, reflecting syntactic and semantic aspects of RC constructions. In a comprehensive review of cross-linguistic differences in attachment preference, Grillo and Costa (2014) raised important issues about syntactic characteristics of experimental stimuli used in prior studies. In fact, they convincingly argue that at least some of the studies contain Pseudo-relative (PR) small clauses (Cinque, 1992). In Spanish, for example, the structure “*que* + Verb phrase” can be either a RC or a PR small clause. There are a number of differences between the two clauses: RCs modify an NP, while PRs are a VP modifier and appear in a more restricted set of contexts (e.g., after perception verbs: (2), adapted from Grillo and Costa, 2014):

(2)
  (a) *Ví al hombre que corría.*                                                                    PR/RC
  I saw the man that ran.
  I saw the man running
  (b) *Viví con el hombre que corría.*                                                            RC only
  I lived with the man who ran

Importantly, there is no PR ambiguity: speakers always adopt a HA interpretation. A large proportion of PR sentences may therefore induce a preference shift from LA to HA (Grillo et al., 2015). Grillo and Costa (2014) recently observed that when controlling for structural characteristics (limiting pseudo-relative availability), speakers adopt an LA preference (Frazier, 1979) across languages, although this preference can be modulated toward HA with long RCs [Implicit prosody hypothesis, Fodor, 1998, 2002; see also Hemforth et al. (2015) for a recent account]. Additional factors affecting RC interpretation, including lexical-semantic information (MacDonald et al., 1994), have been discussed. However, most studies have been conducted with written stimuli, which give us little information about auditory language processing.

Auditory RC comprehension has been studied in a variety of languages such as English (Fernández and Sekerina, 2015), German (Augurzky, 2005), Spanish (Teira and Igoa, 2007), and Bulgarian (Stoyneshka et al., 2010). Most of the studies inserted breaks (e.g., silence) after NP1 or NP2 and presented the resulting isolated (without context) sentences using a two alternative forced-choice task (e.g., “Who was on the balcony?”). Overall, these studies suggest that prosody has asymmetrical effects on disambiguation (Fernández and Sekerina, 2015). A break after NP1 strongly reinforces the pre-existing LA preference, but a break after NP2, elicits a weaker (or altogether absent) modulation toward HA, without overriding the initial LA preference. There is an overall agreement that prosodic breaks act as boundaries (Wagner and Watson, 2010), such as a break after NP1, for example, favors binding between NP2 and the RC. Different accounts hypothesize that breaks act as a “grouping” cue (Informational boundary hypothesis: Clifton et al., 2002), or a disjunction cue (anti-attachment hypothesis: Watson and Gibson, 2005). In addition, as pointed out by Fernández (2007), and also reflected in the available data on ambiguous RCs, some breaks seem to have a stronger effect than others. It is possible that a strong universal LA preference (e.g., Late Closure principle^1^: Frazier, 1979; see also recent arguments by Grillo and Costa, 2014) prevails over prosody. Alternatively, prosody may play a somehow more important role than previously thought, and that is only the case that uncontrolled confounding variables such as lexical-semantic cues and pragmatics (MacDonald et al., 1994; Desmet et al., 2006) introduce noise and mask the effects of prosody in RC auditory studies.

Along these lines, Fernández and Sekerina (2015) pointed out that the aforementioned studies all use semantically deep sentences such as (1), but do not control^2^ for lexical-semantic or pragmatic factors that may influence the participants’ responses. For example, in sentence (1), an actress is more likely to be on a balcony than a maid. To compensate for this issue, Fernández and Sekerina used an auditory-picture matching task with semantically ‘shallow’ English RCs. In their study, semantically shallow sentences described simple geometric shapes (e.g., “*What color is the tip of the triangle that has an umbrella in the middle?*”), to be matched with visual displays, which, contrary to animate NPs, did not require engaging in deep semantic processing as one would with animate NPs. The authors compared the proportion of HA to chance level (HA proportion: 50%), and observed that it decreased significantly (by 24%) when a prosodic break was inserted after NP1, but did not find any significant difference with a break after NP2 (despite a numerical increase of 12%). However, their conclusion was based on the assumption that, without any cues, listeners would be equally likely to pick either interpretation. However, the use of long RCs may favor a HA preference, while short RCs favor a LA preference. In sum, in addition to examining the shift of attachment preference compared to chance level, effect sizes should also be estimated in comparison to a baseline, prosody neutral condition.

Only two studies have implemented a prosody neutral condition: Teira and Igoa (2007) in Spanish, and Fernández (2007) in English. Fernández (2007) recorded three versions for each ambiguous sentence with a break after NP1 (180 ms on average), NP2 (247 ms on average), or no break. In contrast, Teira and Igoa (2007) manually deleted the 500 ms breaks and neutralized f0 to create a “null prosody” control condition^3^. Despite differences in acoustic manipulation, both Fernández (2007) and Teira and Igoa (2007) describe a similar modulation of attachment preference due to the insertion of prosodic breaks, compared to their baseline (43 and 38.8% HA preference, respectively). That is, a weak effect toward HA when a break follows NP2 (48 and 46.5%) and a strong modulation toward LA when a break follows NP1 (16 and 12.7%), compared to the baseline^4^. However, for the swing toward HA interpretation following NP2 breaks the modulation they report comes closer to chance level, but does not actually *reverse* the interpretation to a HA preference.

We created three experimental conditions varying the presence and position of a prosodic break in ambiguous RC sentences. If listeners rely on prosody to facilitate online comprehension, prosodic break placement after *either* NP1 or NP2 should change the interpretation to LA or HA, respectively. To test this hypothesis, we suggest an alternative approach by adequately controlling for lexical-semantic bias. First, we matched NP1 and NP2 in frequency for every sentence. Indeed, word frequency (Bybee and Hopper, 2001; Lau et al., 2008), may influence interpretation: for example, very frequent words may be selected by participants over infrequent ones. Second, we pre-tested our sentences in an additional pool of participants in order to make sure stimuli were indeed perceived as ambiguous. We decided to embed the experimental sentences within a context, to make them part of a story. We reasoned that prosodic effects might reveal themselves more naturally under a realistic context (given that, in real life, sentences are rarely processed in isolation). Finally, we implemented a prosody-neutral baseline, which will allow us to quantify the magnitude and symmetry of the change in attachment preference, so that any trend in either way could be revealed.

Our experiment mainly focuses on the interplay between prosody and syntax, for which interpretation responses are the most informative dependent variable. Following previous accounts of short RC attachment in reading (Fodor, 1998, 2002), we predicted a slight preference for LA in the absence of prosodic cues, thus providing an appropriate baseline to measure the effects of prosody. Crucially, an effect of prosody overriding syntactic preference would be quantified by (a) the relative shift in attachment preference as compared to baseline, and (b) a modulation of this preference compared to chance level (HA 50%). We expected that the position of the prosodic break would introduce a change in attachment preference in either direction. If so, a break after the first noun (NP1) was expected to trigger a LA preference because it would bind the second NP with the RC, while a break after the second noun (NP2) should change this interpretation toward HA because it would first bind the two NPs and then the RC to them. Those predictions are compatible with both anti-attachment and informative boundary hypotheses, as the experimental sentences have a maximum of one 200 ms-pause. Reaction times (RTs) may provide us with some additional information. If listeners truly benefit from a prosodic break to disambiguate the sentences, as the anti-attachment hypothesis predicts, then both break conditions should result with a faster RTs compared to the, putatively more ambiguous, baseline.

## Materials and Methods

### Participants

Twenty native Spanish speakers (9 females, mean age: 23 ± 5 years) volunteered after giving informed consent, in exchange for 10 €/h. All participants had normal or corrected-to-normal vision and no known hearing deficits. One participant was removed from analyses for responding after the time limit on more than 35% of the trials.

### Stimuli

A hundred and six (106) sentences containing attachment ambiguity such as the last sentence in (3) were created along with their context, the first two sentences in (3):

(3) *Este año se puso en marcha una gran campan
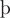
a anticorrupcioìn en la ciudad. Hoy fue el uìltimo diìa despueìs de una semana de acciones. La policía arrestó al protegido del mafioso que paseaba*.
This year the city started a large anti-corruption campaign. Today was the last day of week of actions. The police arrested the protégé of the mobster who was walking.

In order to keep stimuli as ambiguous as possible in the absence of prosodic cues, the RCs inserted in the sentences were shorter than six syllables (based on de la Cruz-Pavía, 2010). All NPs contained between three and five syllables including the determiner to insure rhythmically similar stimuli. Each experimental sentence was preceded by a context, to enhance naturalness and introduce a prosodic rhythm. So far, no study has reported introducing context sentences to improve the ecological value of the experimental stimuli in the auditory domain. A few studies have looked into the effect of context manipulation on RC attachment disambiguation. For example, Desmet et al. (2002) showed that introducing only one referent (NP1 or NP2) in the sentence immediately preceding the experimental sentence influenced attachment preference in an off-line, completion questionnaire, but did not seem to affect reading strategies in the principle on-line measure, using eye-tracking (where sentences that are disambiguated toward NP1 had an advantage regardless of the context). Based on this pattern of results, Desmet et al. (2002) concluded that referential information did not affect initial preference for LA. However, as Grillo and Costa (2014) pointed out, preceding results should be taken with caution because PR availability remained unclear (and uncontrolled for). Our experiment was designed so that the contexts should minimally influence interpretation. The large majority of contexts did not mention NP1 or NP2 as referents (84%), 11% mention both, and only 5% explicitly mention one of the referents.

All sentences, with their contexts, were pre-tested in order to verify their ambiguity. Six participants^5^ volunteered for the pre-test. They were presented with both contexts and sentences in written form and were asked to choose an interpretation. Six sentences that elicited an attachment preference (low or high) of more than 70% in at least one participant were excluded. In addition, following Grillo and Costa (2014), nine sentences that led to a Pseudo-Relative interpretation were removed, leaving 91 sentences (see Appendix I for a complete list).

In order to control for lexical effects, dimensions including phonological neighbors, familiarity, imageability, and concreteness of NP1 and NP2 were measured using *EsPal* (Duchon et al., 2013). While phonological neighbors count is an objective measure (based on the number of substitution, addition, and deletion neighbors in a corpus based on movie subtitles^6^), the other three dimensions (familiarity, imageability, and concreteness) are based on subjective measures collected from a large questionnaire (all measures were collected by Duchon et al., 2013). Mean values from these measures reveal that the words used were judged as familiar, concrete and easy to imagine. Levene’s test revealed that the sample was homogeneous in all three dimensions (*p* > 0.05). Most crucially, an ANOVA with NP as a between-item variable (two levels: NP1, NP2) returned no significant effect of familiarity, phonological neighbors, concreteness, and a marginally significant effect of imageability (see Appendix II for details). These measures were fully available for most (but not all) 91 NP pairs used in the experiment. In addition, word frequency was also calculated from the *EsPal* corpus. Paired *t*-tests revealed no significant difference in log(frequency) between the two lists [*t*(60) = 1.505, *p* = 0.138].

There is evidence that, when reading aloud, Spanish speakers tend to project their interpretation using prosodic contours (de la Cruz-Pavía, 2010; de la Cruz-Pavía and Elordieta, 2015), which raises several issues when recording the materials. First, a neutral version is virtually impossible to achieve with natural speech, creating a baseline problem. Second, breaks in both experimental sentences are usually not equal: breaks after NP2 are substantially shorter than after NP1 (see Fernández, 2007). Finally, in natural speech breaks can be shorter (Fernández, 2007) than the definition of a ‘significant pause’ proposed by Nooteboom (1996) that approximates the duration to 150 ms, or twice as long (e.g., 500 ms in Teira and Igoa, 2007). For these reasons, despite some authors choosing to use unedited sentences from natural recordings (Reyes, 2007), we opted for an acoustic manipulation of naturally recorded stimuli. Two versions of each sentence were recorded using a unidirectional microphone MK600, Sennheiser, and the Audacity software (v. 2.0.3; sampling 24 kHz). For each sentence, a female native speaker of standard Castilian Spanish was asked to read versions (6) and (7) in a natural fashion (“#” indicates a break).

(6) [_S_ [_NP0_ La policía ] [_V P_ arrestó [_NP1_ al protegido # [_PP_ d[_NP2_ el mafioso [_RC_ que paseaba ] ] ] ] ] ]
(7) [_S_ [_NP0_ La policía ] [_V P_ arrestó [_NP1_ al protegido [_PP_ d[_NP2_ el mafioso #[_RC_ que paseaba ] ] ] ] ] ]

Using *Praat* (Boersma and Weenink, 2015), the sentences were examined acoustically and visually to make sure they presented homogeneous intonation. The duration of all breaks, both in context and experimental sentences, was adjusted to 200 ms. Sentences (3) and (4) were cross-spliced at the offset of the preposition (‘*del*’) to create the Baseline condition, without prosodic break. Despite a difference in strategy from Teira and Igoa’s (2007), who deleted the breaks and neutralized f0 from their recordings, the cross-splicing technique has been successfully employed in multiple auditory ERP studies (see Pauker et al., 2011 for an example manipulating prosody). This technique has the advantage of preserving naturalness of stimuli by not manipulating f0, while controlling for NP duration, which has been reported to be longer before a pause (e.g., Fernández, 2007), thus allowing each condition to be directly compared to a baseline. Acoustical measurements on all NPs in all conditions^7^ reveal that NPs were substantially longer [NP1: *t*(92) = 17.5, *p* < 0.001, NP2: *t*(95) = -14.42, *p* < 0.001] and reached a higher f0 maximum [NP1: *t*(92) = 10.15, *p* < 0.001, NP2: *t*(95) = -8.44, *p* < 0.001] when they were followed by a break (see Appendices III and IV). However, those differences were compensated for in the baseline condition, where neither NPs appear before a break, and are therefore shorter and unaccented. All sound tracks were then normalized to ensure equal amplitudes across conditions and stimuli. Resulting sentences were judged natural by the authors as well as three native Spanish speakers with phonetic training. In total, the experimental procedure contained three conditions: NP1 (prosodic break after NP1), NP2 (prosodic break after NP2), and Baseline (no prosodic break at all). All 273 sentences were distributed across three experimental lists (*n* = 91) such as every sentence contributed to each condition and sentences would not be repeated within any list.

### Procedure

**FIGURE 1:**
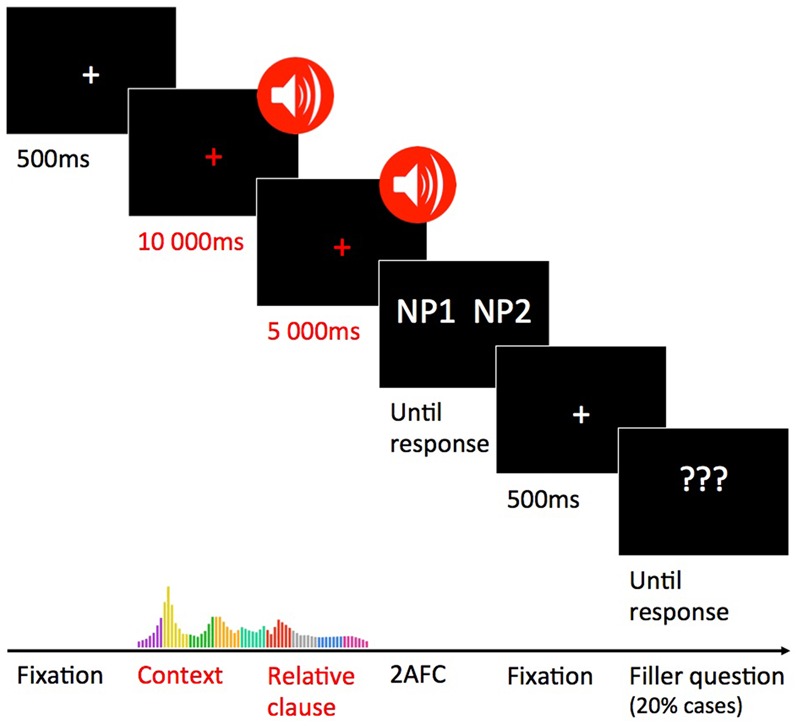
**Schematic representation of the experimental procedure**.

Participants sat on a comfortable chair in a sound attenuated booth; about 60 cm from a monitor where a white fixation cross was displayed on black background. For each participant, all 91 sentences were pseudo-randomized so that the same condition would not be played more than twice in a row. The cross would turn red when the auditory stimulus started. The participants heard each context sentence followed by the corresponding experimental sentence, after which they would be asked to choose between two interpretations (between NP1 and NP2, e.g., “¿‘Quien paseaba?: El protegido/El mafioso”). Teira and Igoa (2007) did not include filler sentences, while Fernández (2007) added comprehension questions on non-ambiguous sentences (30% of trials). Our fillers consisted on two-alternative forced choice filler comprehension questions that where asked about the context sentences at the very end of 20% of trials (see **Figure 1**). Those filler questions ensured that participants attended to stimuli. All of our questions (experimental and filler) were described as comprehension questions, and there was no mention of the ambiguities. We measured the RTs and attachment preference rates.

## Results

On average, the participants responded correctly to 90% (±7) of the filler questions, suggesting that they were paying attention to the experimental sentences.

### Reaction Times

Data were analyzed with a mixed effects regression model using the *lme4* package (Bates et al., 2015) of the R software (R Core Team, 2014). The models were further decomposed into pairwise comparisons of means using Tukey contrast (*multcomp* package: Hothorn et al., 2008). We first analyzed the logarithmic values of the RTs to examine whether prosodic contours would yield differences in processing durations. Break position (none, after NP1 or NP2) was set as a fixed factor. We selected the maximal converging random structure (Barr et al., 2013) which included random intercept for Participants and random slopes for Participants per Condition^8^. The resulting model showed a significant effect of Break position (**Table 1**), suggesting that participants were faster in responding when a break was presented compared to the baseline. Pairwise comparisons revealed that the effect was stronger and more significant when comparing the effect of a Break after NP1 to no Break (*z* = -3.895, *p* < 0.001) than NP2 to no break (*z* = -2.327, *p* = 0.052). There was no significant difference between the RTs elicited by a break after NP1 or NP2 (*z* = 1.714, *p* = 0.199).

**Table 1:** Reaction times: estimates of fixed effects produced by a linear mixed model of Break position with random intercept for participants and random slopes for participants per break position.

Variables	Estimates	Standard error	Df	T	*p*
(Intercept)^∗^	3.49	0.03	18.05	111.27	<0.001^∗∗∗^
Break after NP1	-0.09	0.02	17.88	-3.90	0.001^∗∗^
Break after NP2	-0.05	0.02	17.97	-2.33	0.03^∗^

*Number of observations = 1725, log likelihood = -944.1. ^∗^The baseline (no break) is estimated by the Intercept. Significance codes: ^∗^ < 0.05; ^∗∗^ < 0.01; ^∗∗∗^ < 0.001.*

### Attachment Preference across Conditions

**FIGURE 2:**
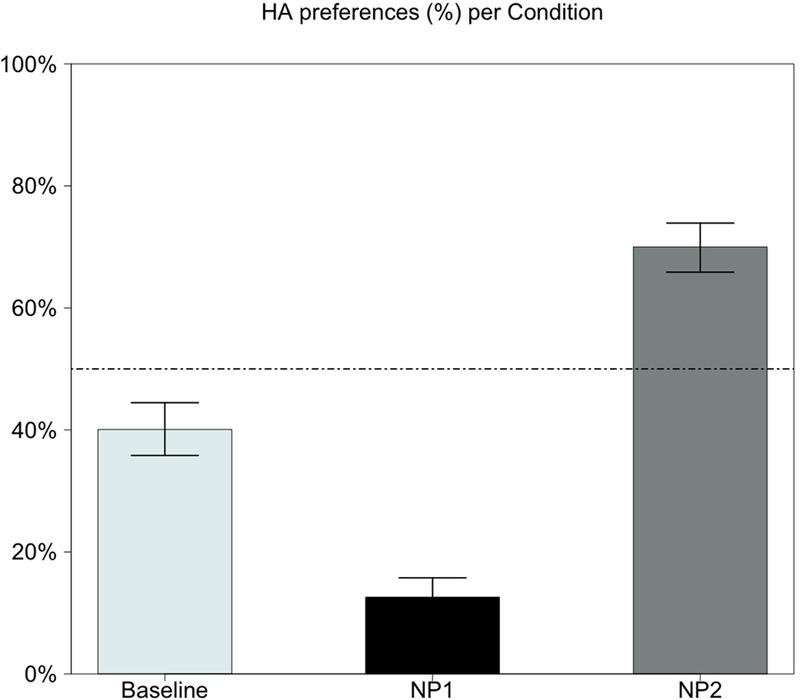
**Modulation of High Attachment preference across Break positions (Baseline: none; after NP1; after NP2).** R (R Core Team, 2014).

Participants’ responses were classified in two categories: HA when participants attached the RC to NP1 (HA), and LA when the RC was attached to NP2. **Figure 2** shows the modulation of HA preference across conditions.

To establish the effect of prosodic break placement on sentence disambiguation, logistic mixed model regression was computed with Break position as a fixed factor and the same random structure as for the RTs (**Table 2**).

**Table 2:** Attachment preferences: estimates of fixed effects produced by a logistic mixed model of break position with random intercept for participants and random slopes for participants per break position.

Variables	Estimates	Standard error	*Z*-value	*p*
(Intercept)	-0.453	0.220	-2.060	0.039^∗^
Break after NP1	-1.971	0.274	-7.198	<0.001^∗∗∗^
Break after NP2	1.597	0.298	5.355	<0.001^∗∗∗^

*Number of observations = 1725, log likelihood = -901.3. Significance codes: ^∗∗^ < 0.05; ^∗∗^ < 0.01; ^∗∗∗^ < 0.001.*

Again, a significant effect for Break position was observed. In the baseline, HA rate was 40% ± 25, suggesting a natural LA attachment preference when no prosodic cues were available to disambiguate sentences. Importantly, the results show that prosodic break placement (both after NP1 and NP2) strongly modulated attachment preference with respect to baseline. When the prosodic break was placed after NP1, HA rate (12% ± 17) dramatically decreased by 28 points compared to baseline (*p* < 0.001). This suggests that the prosodic cue placed after NP1 reinforced the natural preference for the LA interpretation. In contrast, when the break was placed following NP2, HA rate (70% ± 23) increased by 30 points compared to baseline (*p* < 0.001). This result suggests that placing the prosodic break after NP2 inverted the ‘preferred’ interpretation of ambiguous sentences by modulating attachment preference of the RC. The magnitude of the change in either break condition is almost the same, suggesting symmetrical effects of prosodic breaks. These results were confirmed by pairwise comparisons, that showed a significant difference between all pairs (*p* < 0.001).

## Discussion

Auditory studies using ambiguous sentences have shown that prosody may modulate the listener’s interpretation to a certain extent. Specifically, prosodic breaks seem to have asymmetrical effects on sentence disambiguation. Together with the recent developments indicating that there may be a universal parsing preference toward LA (Grillo and Costa, 2014; *contra* Cuetos and Mitchell, 1988), it could be that this preference would take precedence over prosodic cues, consistent with syntax-first approaches (e.g., Late Closure principle; Frazier, 1987). The present study explored the alternative explanation that many factors such as lexical-semantic features may also intervene and bias the results. We suggested that the modest effects of prosody on auditory RC interpretation reported to date might be due to lack of methodological control over some key variables in addition to (or instead of) universal parsing preference of RC structures. Unlike previous studies, stimulus dimensions potentially responsible for noisy and/or artefactual results, such as word frequency, imageability, pseudo-relative availability, and contextual effects, were all controlled for in this report.

Our results show that prosodic cues reinforced the preferred interpretation or reversed it compared to the baseline, but also, for the first time, that listeners actually prefer to attach high (70%) over low, when a break follows NP2. This shift suggests that prosody interacts with syntactic preferences to modulate the interpretation, which is incompatible with a strong “syntax-first” approach. RTs offer some complementary information to the interpretation results. Indeed, RTs were faster when a break followed NP1, though there was only a marginally significant difference between the break-after-NP2 and baseline conditions (as revealed by the pairwise comparisons). These results would be more in line with a global approach to prosody-syntax integration (such as the Informative boundary hypothesis: Clifton et al., 2002; see also Pierrehumbert and Hirschberg, 1990). This approach suggests that listeners seem to weigh out the prosodic cues with previous prosodic boundaries provided by the signal. The Informative boundary hypothesis contrasts with the anti-attachment hypothesis (Watson and Gibson, 2005), which predicts that listeners immediately translate prosodic breaks as a cue not to attach with a previous constituent. However, it is not necessarily incompatible with on-line evidence for immediate integration of prosodic cues using event-related potentials (Steinhauer and Friederici, 2001).

Our study, like Fernández’ (2007) and Teira and Igoa’s (2007) employ short RCs. One should keep in mind that, as evidenced by Clifton et al. (2006), the effects of prosody are stronger when they apply to shorter phrases. and our study employ short RCs. The implementation of a neutral baseline condition was instrumental in evaluating the effect of prosody on sentence disambiguation. Two prior studies have used this approach (Fernández, 2007; Teira and Igoa, 2007). Both reported that prosody could reverse interpretation relative to a baseline (although the statistical evidence confirmed this interpretation only partially). Yet, these studies did not provide evidence for an absolute reversal in interpretation, given that listeners never seemed to prefer a HA over LA interpretation (above and beyond 50%) even in the most favorable conditions. Our results on RC interpretation contrast in that respect, as we demonstrated that listeners do shift their interpretation to a HA preference (70%), when a prosodic break followed NP2. Our methods differ in three major aspects with Teira and Igoa’s and Fernández’s methods: the lexical-semantic controls used to select the stimuli, the use of the cross-splicing method to create the baseline condition, and the choice of fillers. The cross-splicing method (e.g., Steinhauer et al., 1999; Pauker et al., 2011) was employed to prevent possible baseline contamination from the speaker projecting their own interpretation in the production of the materials. However, the proportion of HA responses in our baseline condition (40%) were quite similar to the one in Fernández’ (2007) and Teira and Igoa’s (2007) (42 and 38.8%, respectively). First, this confirms that our baseline material was valid and comparable to theirs, and second, it suggests that the method used for baseline creation (natural or cross-spliced stimuli) has little effect on the participants’ interpretation. Finally, unlike Fernández (2007), our study did not contain additional distractors other than the filler questions. This could have drawn attention to prosodic cues and overestimated their effect. However, this conclusion is unlikely because our effects were larger than Teira and Igoa (2007) who did not include filler sentences or questions at all. Moreover, this unlikely emphasize of attention on prosody would have affected equally the three conditions in a comparable way. Therefore, we suggest the control of lexical-semantic cues seems to be the best candidate to explain the stronger effect of prosodic cues, especially when a break follows NP2, in our study. Therefore, this study not only helps to corroborate the large importance of prosodic cues, but also to establish appropriate controls that help reduce noise in sentence processing studies. Our materials may therefore be used in further studies on RC attachment ambiguities.

Finally, we believe that the novelty of embedding the sentence in a context has helped inducing a more naturalistic-like processing of the materials in the experiment. We argue that the insertion of context sentences gave some legitimacy to the ambiguous RCs. Instead of being presented in isolation and seemingly irrelevant, the experimental ambiguous sentences could be integrated in a story. At this point, however, this must remain only a speculation as we did not measure the effects of breaks in sentences without a context. We base this speculation on the fact that participants were given the opportunity to familiarize with the speaker’s rhythm and voice’s intonations by short stories preceding the experimental sentences for which prosodic modulations were crucial. We hypothesize that speakers implicitly convey one correct interpretation using prosodic cues such as breaks. In turn, listeners are experts in extracting these cues in order to help build adequate syntactic structures and extract meaning, making effective communication generally possible. This may reflect in part the high flexibility that listeners have regarding the great variability in accents or cadences between speakers. On the other hand, in natural scenarios listeners use context to narrows the possibilities of interpretations of the incoming speech to avoid picking the ‘wrong’ interpretation (Gilboy et al., 1995). Though this may also be an important strategy in natural speech, in our experiment contexts were designed not to bias participants toward either interpretation, in order to isolate any effects of prosody. However, the fact that we have obtained a stronger effect of prosodic cues, even compared to Fernández (2007) and Teira and Igoa (2007) who also implemented a baseline, when controlling for lexical-semantic factors suggests that these factors matter, perhaps more than syntactic preference or prosodic cues, during communication. In addition, written studies indicate that context (semantic and pragmatic) has a dramatic effect on RC attachment preferences (Gilboy et al., 1995). Future research could focus on the interplay between prosodic breaks and semantic features, and address whether prosody can still have an impact on interpretation in the face of (or when pitched against) lexical-semantic and contextual expectations.

In sum, our results replicate numerous studies showing that listeners generally prefer to attach low (e.g., Fernández, 2007; Teira and Igoa, 2007) when listening to RC ambiguous sentence in the absence of conflicting cues (40% HA). Listeners also benefit from cues that reinforce this preference (shorter RTs when a break is after NP1). However, this initial preference is not inalterable: interpretation results show that after controlling for lexical-semantic factors and pseudo-relative availability, participants are more sensitive to prosodic cues than previously reported. In fact, even a break after NP2 does not only modulate their interpretation in toward HA relative to baseline, but also beyond a 50% HA preference threshold.

## Ethics Statement

The protocol of the study was approved by the Clinical Research Ethical Committe (Comité Etico de Investigación Clinica)-Parc de Salut Mar, from the University Pompeu Fabra.

## Author Contributions

LF was responsible for designing the experiment, creating the stimuli, testing the participants, analyzing the behavioral data, and writing the article. She was supervised by EB and SS-F. EB reviewed the stimuli and was present during the recording of the materials. He also closely supervised testing, and participated in writing the article. SS-F gave the final word for the appropriateness of the stimuli, and closely supervised the analyses of the results. He also gave valuable input regarding the theoretical impact of the study, and participated in writing the article. All authors contributed to all for criteria for authorship as defined by Frontiers.

## Conflict of Interest Statement

The authors declare that the research was conducted in the absence of any commercial or financial relationships that could be construed as a potential conflict of interest.

## Footnotes

1 Attach new items into the phrase that currently processing (= LA preference in our case).

2 To our knowledge, none of the auditory RC studies report having controlled for parameters such as frequency, imageability, familiarity, nor pre-testing their materials to ensure ambiguity.

3 The authors do not specify which version (break after NP1 or NP2) they used to create the unique “null prosody” condition. Hence, there may be differences in lengthening that could not be neutralized and may lead to one or the other interpretation.

4 It should be noted that neither of these studies provide full conclusive statistical support of the effects (Rosnow and Rosenthal, 1989; see also Nieuwenhuis et al., 2011). Fernández (2007) performed separate ANOVAs for each condition pairs, instead of submitting the three conditions (break after NP1, NP2, no break) as a factor (three levels) and then performing *post hoc* comparisons with appropriate multiple comparison corrections. Hence, we believe, their analysis increased the risk of type I error. Teira and Igoa (2007) based their conclusion only on the significance of the ANOVA prosody factor (three levels: break after NP1, after NP2, no break) without *post hoc* pair-wise comparisons. It is thus difficult to infer that the effect of condition was indeed present between all pairs of conditions. More information (such as standard deviations) would be needed in order to assess whether the modulation from Baseline to HA in the NP2 condition is statistically significant.

5 The participants who volunteered to the pre-test did not take part in the actual experiment.

6 See EsPal website (http://www.bcbl.eu/databases/espal/) for definitions and Duchon et al. (2013) for a description of corpus and subjective measures collection.

7 NP2 are slightly longer in duration than, because they comprise more phonemes (Mean = 7.9 vs. 7.7 phonemes).

8 The model did not converge when including Item as a random intercept or random slopes for Item per Condition.

## Abbreviations

HA: high attachment
LA: low attachment
NP: noun phrase
RC: relative clause
