# Supplementary material for: Searching High and Low: Prosodic Breaks Disambiguate Relative Clauses
Source: Front Psychol. 2017 Feb 1;8:96. doi: 10.3389/fpsyg.2017.00096 (PMC5285353; doi:10.3389/fpsyg.2017.00096)
Supplement: Supplementary file 1 [file Data_Sheet_1.docx]

**Appendix I**

List of Spanish sentences and their context

1. Era una gran cena. Había personas de todas partes y todas las profesiones, todos conversando y riendo. Pero nadie habló al colega del arqueólogo que vino.
2. Estaba en la biblioteca estudiando, pero había una mujer hablando con todo el mundo, que era muy molesta. Ella buscaba a la hermana de la abogada que leía.
3. Mi hermano Mario y yo, fuimos de vacaciones a Roma. Recuerdo que compró postales para toda la gente que conocía. También escribió al criado del escultor que se aburría.
4. Hacía mucho tiempo que no veía a mi hermana. Por eso, la visité la semana pasada, pero no era buen momento... porque mi hermana estaba consolando a la compañera de la niña que estudiaba.
5. Ayer me invitaron a casa de un compañero de clase, que es muy rico. Cuando llegamos a la entrada, alguien abrió y nos hizo pasar. Mi madre saludó al mayordomo del señor que emigró.
6. Ahora vivo en la ciudad, pero en Navidad me fui a mi pueblo. Vi a todo el mundo, a los amigos, a los vecinos, a todos los habitantes! Pero no reconocí a la empleada del barbero que se casó.
7. Pedro es muy activo en política. Una vez, fue a un debate que emitían en directo por televisión. Pero ya no puede ir a ninguno más Porque insultó al asistente del político que temblaba.
8. La actriz no se separaba nunca de su hija. Hasta se llevaba una niñera para cuidarla mientras trabajaba. La mujer ayudó a la hija de la actriz que se levantó.
9. Mi mejor amigo es un romántico. Escribe cartas, poemas... Y parece que le encantan los amores imposibles. Amaba en secreto a la esposa del autor que roncaba.
10. Este año se puso en marcha una gran campaña anticorrupción en la ciudad. Hoy fue el último día después de una semana de acciones. La policía arrestó al protegido del mafioso que paseaba.
11. Como cada día, la abuela iba al mercado a comprar productos frescos. Al salir de la carnicería, perdió su cartera. Alguien llamó a la abuela del carnicero que se marchó.
12. La familia se reunió para pasar el fin de semana en casa de mi hermano. Todo el mundo era bienvenido así que Un viejo amigo visitó a la amiga del verdulero que cocinaba.
13. Hoy fui a una rueda de prensa donde presentaron una nueva revista que tiene que salir pronto. Clara fotografió al entrevistado del periodista que se fue.
14. Como cada día iba a comprar flores para mi novio,( y por ello )empecé a hacerme amiga del florista. Hoy me han presentado a la esposa del florista que cantó.
15. Perdimos la pista de nuestra hermana durante 2 semanas. Por eso, pedimos ayuda a un detective muy famoso que la encontró 2 días después. Mi hermano abrazó al empleado del detective que se ofendió.
16. Cuando un atleta gana un premio, los medios no le dejan en paz. Precisamente esto es lo que pasó en la competición de ciclismo. Llamaron al patrocinador de la ganadora que suspiraba.
17. Trabajo en una compañía de valet. Ayer fuimos a tomar algo con todo el equipo. Me presentaron a la compañera de la bailarina que bebía.
18. Antes trabajaba en una empresa de mudanzas. Solía hablar con los clientes para planificarlo todo. Así encontró a la asistente del senador que se mudó.
19. Conozco una isla donde los habitantes vivían en autarquía hasta 1990. Muchos eran pescadores, marineros,... pero también había una maestra, y una doctora. Todos se conocían! Los vecinos saludaban a la doctora del marinero que comía.
20. El fin de semana pasado, fui de fiesta con un amigo que pertenece a una familia adinerada. Por la noche fuimos a su mansión y estuvimos haciendo mucho ruido! Molestamos al esposo de la marquesa que se despertó.
21. El actor y su primo son muy parecidos. Así que los paparazis a menudo les confunden. Una vez que paseaban juntos, al separarse, el periodista siguió al primo del actor que silbaba.
22. Mi hermano pequeño necesita la ayuda de una logopeda para mejorar su pronunciación. Como es muy simpática, nos hicimos amigas y me presentó a su familia. Nunca ví a la prima de la logopeda que regresó.
23. Hacen un programa en la televisión donde reúnen a personas que no se veían en años. Ayer invitaron al padre del astrólogo que se animó.
24. Antes de que los astronautas se vayan a una misión, organizan una gran comida con toda la familia y amigos. Pero la última vez, olvidaron invitar al suegro del astronauta que estornudaba.
25. A mi madre le encanta la Navidad. En diciembre, ofrece turrón a todo el mundo. También se lo regaló a la empleada del panadero que murmuraba.
26. Suelo acompañar a mi novio cuando corre un maratón. Pero solo voy a mirar, porque es muy cansado. Hay muchos corredores que necesitan andar un poco para recuperarse. La última vez, estuve siguiendo el maratón con el ahijado del atleta que caminaba.
27. Vivía en el Pirineo, cerca de una pequeña estación de esquí donde nos conocíamos todos. Este invierno volví, y otra vez vi a mucha gente del pueblo! Encontró a la nieta del constructor que esquiaba.
28. Esta noche estuve mirando un documental sobre el toreo Las periodistas entrevistaron al abuelo del torero que se afeitaba.
29. Ayer estaba buscando a la jefa del personal para una entrevista. Pero como no la había visto antes, me equivoqué de persona. me presenté a la suegra del programador que se rio.
30. Como fue robada una gran cantidad de dinero del banco, los responsables de la sede empezaron a preguntar a todos los empleados,.. hasta interrogaron a sus familias! Intentaron llamar a la cuñada del consultor que huyó.
31. Los niños que estaban jugando con petardos, fueron los responsables de incendiar el campo! Los niños siguieron al amigo del huérfano que se quemó.
32. Delante de casa había un gato subido en un árbol que no sabía cómo bajar. Por suerte encontraron una mujer cuyo yerno era bombero, así que juntos le salvaron. Todo el mundo felicitó a la madrastra del bombero que aplaudía.
33. Estos días que hace más calor voy a la playa. Allí, siempre me encuentro a gente conocida! Ayer me encontré al abogado del vendedor que nadaba.
34. Me gusta mucho pasear por los jardines de Montjuic. Me parecen muy bonitos. Ayer felicité al jardinero del paisajista que trabajaba.
35. En el mundo del deporte, para combatir la corrupción y el dopaje, hay que tomar a veces decisiones radicales. El club rechazó al entrenador del ciclista que engañaba.
36. Se celebró un juicio tras el terrible accidente. Mucha gente asistió a declarar El juez escuchaba al compañero del estudiante que conducía.
37. Habían rumores de que el director de la fábrica estaba explotando a sus empleados. No sabíamos si fiarnos de ellos, ya que era un hombre muy apreciado en la comunidad. Pero al final despidieron al director del obrero que protestó.
38. En la última convención humanitaria que celebramos, invitamos a una monja India conocida por ayudar a construir un orfanato. Al final de la convención, todos fueron a un restaurante para celebrar su gran labor. Mientras cenando, encontré al intérprete de la monja que pagó.
39. El cartero estaba enfermo, pero siempre se olvidaba de tomar sus medicamentos. Su esposa estaba bastante preocupada. Por eso llamó al farmacéutico del cartero que se sentó.
40. El cura tenía problemas de visión, así que mientras recitaba el sermón no podía ver a nadie! Al final fue al oftalmólogo y le pusieron unas gafas. La parroquia agradeció al oftalmólogo del pastor que se alegró.
41. Mi hermano ha empezado a estudiar letras y filosofía en la universidad. Le gusta mucho una asignatura que es impartida por un profesor argentino y su asistente. Mi hermano admira al estudiante del filósofo que se casó.
42. En breve empezará la campaña para las elecciones presidenciales. Los medios de comunicación, como siempre, se van a poner como locos. Ayer invitaron a la televisión al psicólogo del candidato que se presentó.
43. En la editorial donde trabaja Luis, han robado un manuscrito. Todo el mundo está buscando al culpable. Luis acusó al secretario del editor que se enojó.
44. Anna fue a visitar un amigo que está en el hospital, en el servicio de neurología. Estaba buscando al neurólogo del paciente que se alejó.
45. Mi novio y yo ganamos un concurso para ir a ver un partido del Barça. Después pudimos visitar al equipo en los vestuarios. Mi novio saludó al fisioterapeuta del futbolista que vino.
46. En Finlandia es obligatorio hacer el servicio militar. Tengo un amigo que lo hizo hace unos años. Odiaba al capitán del soldado que se salvó.
47. El niño vivía con su abuela. Como se preocupaba de la educación del chico, invitó al maestro a comer en casa. La abuela miraba al maestro del chico que se hartó.
48. Bruno trabaja para una empresa de transporte. Allí tienen duchas y camas para los que tienen que trabajar de noche. Bruno buscaba al jefe del camionero que se afeitaba.
49. Ayer fui a ver una película de James Bond. Era muy divertida, pero me sobresalté en una escena! Es que no había visto al espía del traidor que disparó.
50. El fin de semana pasado, fui al teatro. Me gustó mucho la obra! No deje de mirar a la pareja del comediante que interpretaba.
51. El embajador es un hombre muy simpático, pero contrató a un mayordomo muy orgulloso. Ignoró durante 2 días al invitado del embajador que firmaba.
52. Me gustan mucho las historias de piratas. Pero el otro día vi una película un poco antigua, que no era muy buena. Solo presté atención al loro del pirata que cayó.
53. Hace una semana llevé a mi sobrino al circo, porque nunca había ido. La verdad es que a mí también me impresionó bastante! Todos admiraban al tigre del domador que subía.
54. Un desconocido vino a ver un ensayo de la compañía del circo. Creo que estaba interesado en una chica... El extranjero llamó a la prometida del acróbata que se giró.
55. El poeta escribía unos poemas muy hermosos. Parece ser que se inspiraba en una musa guapísima. Los jóvenes amaban a la musa del poeta que vagaba.
56. El libro que estoy leyendo tiene mucha acción. Ahora casi he llegado al final... En el último capítulo la momia amenazó al enemigo del aventurero que escapó.
57. La ciudad de Barcelona se puede visitar con un guía que explica la historia de los monumentos. El camarero saludó al guía del turista que se perdió.
58. Antes, María trabajaba en un restaurante, así que conoce mucha gente de la profesión. Conocía al proveedor del restaurador que escalaba.
59. A mi hermana y a mí, nos gusta la natación. Cuando podemos, vamos a ver a competiciones. Además, mi hermana ama al competidor del nadador que ganó.
60. Hace tres semanas, fui a la ópera con mi novio. Aunque no soy una experta, creo que no era demasiado buena la obra El público aplaudió al solista del coro que tocaba.
61. Estoy mirando una serie en la televisión de fantasía. En el último episodio, los magos hechizaron al propietario del dragón que soñaba.
62. El emperador tenía siempre muchas visitas. Además, era conocido por hacer fiestas maravillosas. Cuando la princesa entró, los sirvientes agasajaban al consejero del emperador que disfrutaba.
63. El golfista tiene una admiradora que siempre le sigue cuando tiene una competición. A veces hasta se acerca a la pista para abrazarle. El espectador gritó al hincha del golfista que se enfadó.
64. Daniel estaba visitando un museo, pero era tan grande que se perdió! Preguntó al seguidor de un conservador que se mofó.
65. Muchas veces cuando mi vecino se va de vacaciones o no está en casa, me encargo de dar de comer a su gato Acaricié al gato del vecino que enfermó.
66. Cuando era pequeña, siempre quería ir a buscar pan porque podía comprarme caramelos con el resto del dinero que sobraba. Así conocí al nieto de la panadera que se marchó.
67. EL quiosco fue robado por tercera vez en un mes! Los vecinos le dijeron a la policía que tenían que encontrar al responsable. El policía buscó al ladrón de la quiosquera que cojeaba.
68. En la escuela realizaron un simulacro de incendios. Todos tenían que esperar en una sala de seguridad Autorizaron la salida al auxiliar de la profesora que se estresó.
69. En mi barrio todos tienen perro. A mí me gusta mucho jugar con ellos. Conocí al perro del hippy que se fugaba.
70. Cada vez que cambio de país, tengo que hacer tareas administrativas muy aburridas. La última vez que estuve en Canadá, me pidieron muchos papeles. Solicité el documento a la secretaria del burócrata que se sofocó.
71. La excursionista se presentó en la barbacoa con sus amigas. Había una chica que parecía muy triste. Consoló a la conocida de la excursionista que salió.
72. Fui a una exposición multimedia que me resultó muy inspiradora! Nunca había visto nada similar. Quería hablar con los artistas. Esperé a la salida al fotógrafo del artista que entró.
73. Después del éxito de la exposición, todos fueron a tomar una copa de cava. Hicieron muchas preguntas a la traductora del pintor que temblaba.
74. Víctor tenía que cambiar la hora de su reunión con el ejecutivo de una compañía aseguradora. El asistente le puso en contacto con el ejecutivo Víctor agradeció la ayuda al telefonista del ejecutivo que bromeaba.
75. Un hombre muy raro que se acercó a uno de los concejales, fue arrestado por posible terrorista. Acusó de mentiroso al guardaespaldas del concejal que vaciló.
76. En mi escuela había dos chicas gemelas, tan parecidas que toda la gente las confundía. Yo hablaba mucho a la melliza de la alumna que devoraba.
77. Al senador no le gustaba el gobierno actual, ya que había mucha corrupción y él no quería formar parte de eso. Pero, muy a su pesar tuvo que apoyar al subordinado de la ministra que manipulaba.
78. Cada fin de semana, voy a escalar. La última vez fue muy difícil la ascensión porque se produjeron desprendimientos de rocas . Avisé del peligro al instructor del escalador que se hirió.
79. Ramón era un comercial que vendía relojes a domicilio. Al final cambió de productos porque no acabó bien con su proveedor. Ramón defendió a la aprendiza del relojero que estafaba.
80. Solía ver una serie de investigación que se acabó ayer. Todos los malos fueron arrestados y en el último episodio se celebró el juicio. El jurado condenó al cómplice del asesino que se lamentó.
81. Las compañías aseguradoras suben el salario a los que cierran más contratos para motivarlos. Eso le dije al responsable de la agencia que competía.
82. Miguel es un peluquero que trabaja a domicilio con su ayudante. A veces los clientes le llaman en el último minuto, así que tiene que coger un taxi. El taxista conoció al ayudante del peluquero que volvía.
83. Mi tío solicitó ayuda a un arquitecto antes de diseñar su casa. El jueves pasado estuvieron hablando de los planos. Estaba esperando a la asistente del arquitecto que sonreía.
84. Javier viaja mucho. Como no tiene dinero, trabaja en granjas a cambio de comida y cama. Javier habló al jornalero del agricultor que fumaba.
85. En el barrio hay un hombre muy pesado que habla con todo el mundo. Su ahijado trabajaba en una churrería, así que pasaba mucho tiempo allí. Un amigo empujó al padrastro del churrero que mentía.
86. Voy a una frutería donde los productos son muy buenos y baratos. Me hice amigo de la gente que trabaja allí. El otro día encontré al gemelo del frutero que balbucía.
87. Mis abuelos quieren aprender a utilizar un ordenador, por eso se compraron uno. Pero siempre tienen problemas y necesitan a alguien para ayudarlos. Llamaron al técnico del informático que se resfrió.
88. El escritor tiene una familia muy grande, que está muy unida. Cuando alguno tiene pareja, tiene que presentársela a todos! La familia invitó a la pareja del escritor que paseaba.
89. En la universidad fui a una conferencia muy interesante sobre los aspectos económicos del mundo actual. Quería hablarle al socio del economista que esperaba.
90. La pareja había reservado mesa en un restaurante de gastronomía típica catalana, para celebrar su cumpleaños. Pero no fue muy bien la cena. El camarero ofendió a la novia del ingeniero que cenaba.
91. El empresario quería cerrar la fábrica de azúcar, pero los empleados, después de meses de manifestaciones, le hicieron cambiar de opinión. El sindicalista felicitó al empleado del empresario que hablaba.

**Appendix II**

Summary of word characteristics: Mean(SD) values for phonological neighbors, familiarity, imageability and concreteness for NP1 and NP2 from Es-Pal (citation) values, as well as One-way ANOVA results.

|  | **NP1** | **NP2** | **Items (/182)** | ***F*** | ***p*** |
| --- | --- | --- | --- | --- | --- |
| **Phonological neighbors** | 8.22(.97) | 6.8(1.35) | 173 | 1.371 | .243 |
| **Familiarity** | 5.61(.13) | 5.48(.11) | 104 | .759 | .386 |
| **Imageability** | 5.31(.12) | 5.58(.11) | 102 | 2.951 | .089 |
| **Concreteness** | 5.39(.10) | 5.56(.09) | 105 | 1.661 | .200 |

**Appendix III**

Pitch (f0) contours in each test sentence: *A*. break after NP1, *B*. break after NP2, and *C*. cross-spliced version of the first two.

A.

(…) al protegido # del mafioso que paseaba.

B.

(…) al protegido del mafioso # que paseaba.

C.

(…) al protegido del mafioso que paseaba.

**Appendix IV**

Summary of acoustic characteristics for NP1 and NP2 for each of the two experimental conditions (baseline values are in **bold**): Mean(SD) values for duration in ms and fundamental frequency in Hz,: Break after NP1 – Break after NP2.

|  | **Break after NP1** | | **Break after NP2** | |
| --- | --- | --- | --- | --- |
|  | **NP1** | **NP2** | **NP1** | **NP2** |
| **Maximum F0** | 285(28) | **252(15)** | **245(21)** | 305(61) |
| **Duration** | 526(113) | **542(96)** | **395(116)** | 429(90) |
